# Percutaneous fully endoscopic surgical management of the ruptured epidural catheter: Rescue of the novice anesthesiologist from his dilemma

**DOI:** 10.3389/fsurg.2022.915133

**Published:** 2022-10-11

**Authors:** Weijun Kong, Qian Du, Zhijun Xin, Guangru Cao, Dexing Liu, Yiyong Wei, Wenbo Liao

**Affiliations:** ^1^ Department of Orthopedic Surgery, The Second Affiliated Hospital of Zunyi Medical University, Zunyi, China; ^2^ Department of Orthopedic Surgery, The First Affiliated Hospital of Zunyi Medical University, Zunyi, China; ^3^ Department of Anesthesia and Perioperative Care, The Affiliated Hospital of Zunyi Medical University, Zunyi, China

**Keywords:** rupture of epidural catheter, percutaneous, epidural nerve block, epidural analgesia, fully endoscopic technique, epidural catheter

## Abstract

**Background:**

Epidural nerve block and analgesia are basic anesthetic techniques for anesthesia. Epidural catheter rupture and partial retention are adverse events and rare complications of epidural catheterization technique. The probability of occurrence when applied by novice doctors is high. Removal of the residual catheter by conventional surgery causes more trauma and bleeding, slows recovery, and may causes medical disputes. We hypothesized that percutaneous spinal endoscopy a safe and effective remediation technique. This study was to analyze the efficacy and safety of removing the residual dural catheter by a percutaneous full-endoscopic technique(PFET) and discuss the clinical technique and precautions.

**Methods:**

This was a retrospective analysis of 7 patients with ruptured epidural catheters treated in our department from October 2015 to October 2019 using the PFET to remove the remaining epidural catheter. The operation time, intraoperative bleeding volume, surgical complications, and neurological symptoms before and after surgery were recorded. The Self-Rating Anxiety Scale (SAS) was used to assess the anxiety level of the anesthesiologist and the patient before and after the catheter removal operation, and the postoperative low back pain VAS score was recorded.

**Results:**

The remaining epidural catheter was successfully removed from all 7 patients. The operation time was 54.14 ± 14.45 (32–78) minutes, and the intraoperative blood loss was 9.134 ± 3.078 (5–15) ml. There were no cases of dural damage, cerebrospinal fluid leakage, sensorimotor dysfunction of the lower limbs, or bowel dysfunction. The anxiety symptoms of the patient and the anesthesiologist disappeared after removal of the residual epidural catheter. The patients' postoperative back pain VAS score was 0 to 2 points.

**Conclusion:**

PFET is a safe and effective minimally invasive technique for removing residual epidural catheters. It causes less trauma and less bleeding, allows a faster recovery. It does not affect the recovery of patients from other surgical operations and reduces both medical risks and medical costs. At the same time, it avoids or reduces the occurrence of medical disputes and eliminates the pressure on novice anesthesiologists regarding similar adverse events.

## Introduction

Epidural puncture is a basic operation technique for anesthesia and is one of the most effective and safest methods for analgesia in labor and delivery, with an efficacy of over 95% (1, 2). At present, the continuous epidural block method is a basic technique for anesthesia that it is widely used, especially in county-level hospitals, in patients with controlled analgesia, chronic low back pain, labor pain, etc. (3, 4). Related research has shown that the incidence of catheter rupture during epidural anesthesia is 0.1%–0.3% (3, 5). There are many reasons for epidural catheter rupture and partial retention, the most common cause of epidural catheter rupture is insufficient operation experience in novice doctors (6). Of course, there are other special conditions, such as catheter quality problems, uninspected catheters with damage, patients with ligamentous calcification of the ligamentum flavum or hypertrophic arthritis, catheter clipping and trimming during the operation, and rarely, epidural catheter knotting (5–8).

At present, there is no unified opinion regarding the treatment of catheter breakage during epidural anesthesia or analgesia (1, 3). A piece of ruptured catheter in the epidural space is a foreign body, which will cause related inflammatory hyperplastic reactions, the formation of a foreign body granuloma, and the occurrence of related nerve compression symptoms and signs (8, 9). Therefore, most scholars advocate removing the broken catheter as soon as possible to reduce the risk of surgery and iatrogenic injury to the patient (9, 10). Conventional surgery requires removal of the lamina and some of the articular process, destroying stability of the spine. The surgical trauma is extensive, and the postoperative recovery is slow. Even auxiliary pedicle screw internal fixation may be required, which significantly increases the medical cost and leads to medical disputes. At the same time, the anxiety of anesthesiologists has significantly increased, which may seriously affect their practice. Percutaneous full-endoscopic technique (PFET) is currently the standard technique for the treatment of degenerative diseases of the lumbar spine (11). It is less invasive and costly, allows a quicker recovery, and does not destroy the stability of the spine (12). This may be an effective remedy for removing a ruptured epidural catheter. In this study, 7 cases of epidural catheter rupture and partial retention were successfully treated using a percutaneous endoscopic technique to avoid the occurrence of medical disputes, and the technical operation and experience are discussed below.

## Materials and methods

### General information

Seven patients with epidural catheter rupture were treated at our hospital from October 2015 to October 2019, written informed consent was obtained from all individual participants included in the study, and the study protocol was approved by the Ethics Committee in The Second Affiliated Hospital of Zunyi Medical University. There were 3 males and 4 females, aged 45.43 ± 17.74 y (24–68 y). One case occurred at our hospital, and the remaining 6 cases were from different primary county hospitals. All patients underwent the operation at the local hospital. The anesthesiologist determined that the epidural catheter was broken and informed the patient and his or her family that the endoscopic technique was needed to remove the residual epidural catheter. The patient was transferred to our hospital for spinal endoscopy to remove the residual catheter. Before the patient was admitted to our hospital, the author fully communicated with the anesthesia operator to determine the puncture gap, the direction of catheter implantation, and the length of residual catheter. Before the operation, the lumbar spine was examined by x-ray and MRI and the lumbar puncture space was examined by CT to rule out lumbar spine lesions. The epidural space is located in the lumbar segment. There were 3 cases of cesarean section, 2 cases of lower extremity surgery, and 2 cases of pelvic surgery. Catheter rupture occurred during catheterization in 3 cases, during successful needle retraction in 3 cases and after extubation in 1 case.

### Surgery

**Figure 1:**
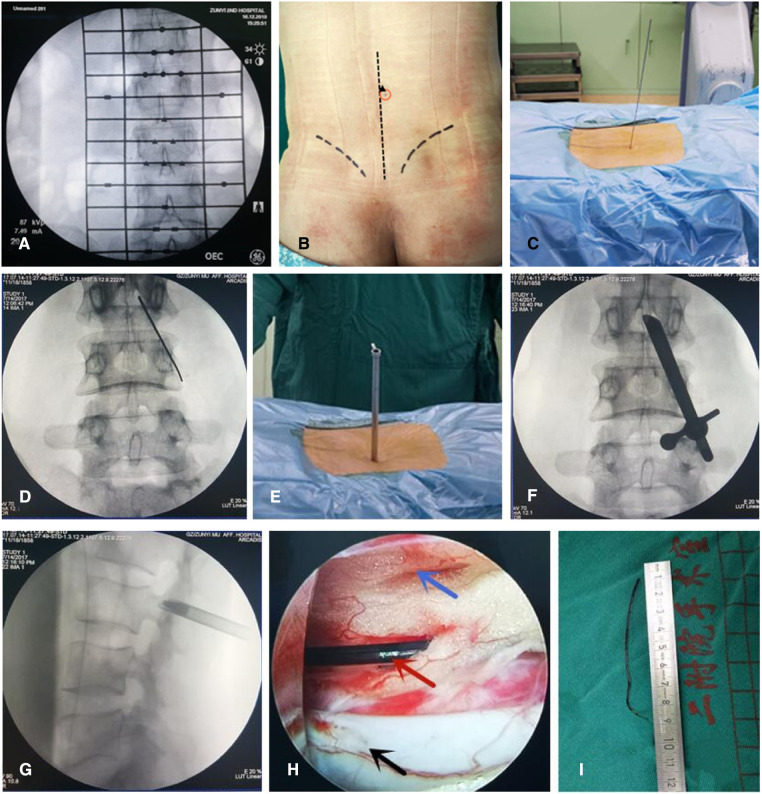
Typical case: Female, 26 years old, cesarean section, lumbar 2-3 epidural space cephalic catheter rupture and residual epidural catheter. (**A,B**) x-ray was used to locate the puncture gap and mark the puncture point; (**C**) a puncture guide needle was inserted at the marked point; (**D**) x-ray fluoroscopy was used to determine the correct position of the guide needle; (**E**) a working sleeve was placed; (**F,G**) x-ray fluoroscopy was performed to ensure that the working sleeve was positioned correctly and that the depth was appropriate; (**H**) The residual epidural catheter was clearly visible under the endoscopic vie; (**I**) Remove the remaining epidural catheter completely.

In one case, the patient was treated immediately at our hospital. The patient underwent surgical anesthesia for bladder stone removal. The epidural catheter was ruptured during the catheterization process. Surgical anesthesia was replaced with general anesthesia. Then, the prone position of the patient was changed, and the puncture point was selected for endoscopic surgery according to the positioning point of the anesthesia operator, the direction of tube placement, and the length of the residual catheter. That is, the anesthesia puncture point was avoided, the head end of the catheter toward the center of the spinal canal was located, the endoscopic operating system was placed, and the remaining epidural catheter was completely removed. The remaining 6 patients underwent surgery under general anesthesia. After satisfactory general anesthesia with intubation was achieved, the patient was placed prone on a fluoroscopic carbon fiber surgical bed, with hip flexion and knee flexion to achieve proper posterior lumbar protrusion. while considering the history of epidural puncture. X-ray was used to locate the puncture gap (Figure 1A) and mark the puncture point (Figure 1B). Conventional disinfection was performed, a sterile towel sheet was spread over the area, and a puncture guide needle was inserted at the marked point (Figure 1C). X-ray fluoroscopy was used to determine the correct position of the guide needle (Figure 1D); an expansion rod was implanted through the guide needle, and a working sleeve was placed (Figure 1E). Then, x-ray fluoroscopy was performed to ensure that the working sleeve was positioned correctly and that the depth was appropriate (Figure 1F-G). The endoscopic operating system was placed, and the operation was begun under continuous saline irrigation. The direction of the working cannula was adjusted to clearly reveal the epidural space and explore the left and right sides from the center to find the remaining epidural catheter (Figure 1H). The residual ruptured epidural catheter was removed (Figure 1I). Radiofrequency hemostasis was performed, the operating system and working sleeve were removed, one needle was used to suture the skin incision, the sterile dressing was covered, and the operation was ended.

### Postoperative treatment

Eight hours after the operation, the patient was placed on supine rest; vital signs were monitored by electrocardiography, and lower limb sensory-motor function and active lower limb functional activities were observed. If permitted by the patient's other surgical conditions, he or she could wear a waist support to get out of bed and walk on the same day. According to individual adaptation, the amount of activity was gradually increased, and the patient returned to normal daily activities.

### Observation indicators

(1) Basic information: the patient's age, sex, anxiety score and surgical site before surgery; the experience level and self-rating anxiety score (SAS) of the responsible anesthesiologist. There are 20 items in the SAS and the point is 1 to 4 (Table 1). The total points are the sum the 20 items. And the subject is considered normal, mild anxiety, moderate anxiety, and severe anxiety while the total points are <50, 50–60, 61–70, and >70, respectively.
(2) Intraoperative observations: the operation time, intraoperative blood loss, and intraoperative complications.
(3) Postoperative observations: wound healing, neurological function, anxiety score, postoperative low back pain VAS score.

**Table 1:** The 20 items in SAS. The point of each item was 1–4.

I feel more nvrvous and anxious than usual	I feel afraid for no reason at all
I get upset easily or feel panicky	I feel like I’m apart and going to pieces
I feel that everything is all right and nothing bad will happen	My arms and legs shake and tremble
I am bothered by headaches neck and back pain	I feel weak and get tired easily
I feel calm and can sit still easily	I can feel my heart beating fast
I am bothered by dizzy spells	I have fainting spells or feel like it
I can breathe in and out easily	I get feelings of numbness and tingling in my fingers / toes
I am bothered by stomach aches or indigestion	I have to empty my bladder often
My hands are usually dry and warm	My face gets hot and blushes
I fall asleep easily and get a good night’s rest	I have nightmares

### Statistical analysis

This study used SPSS 22.0 statistical software to process the collected data. The measurement data are presented as the mean ± standard deviation. Pre- and postoperative anxiety scores were compared by repeated measures analysis of variance. The significance level of the difference was set at *P* < 0.05.

## Results

All 7 patients in this group underwent successful removal of the epidural catheter and didn't lead to medical disputes. It was confirmed by surgery that the tail end of the residual tube was located in the spinal canal; the operation time was 54.14 ± 14.45 (32–78) minutes, and the intraoperative blood loss was 9.134 ± 3.078 ml (5–15 ml). There were no cases of damage to the dura mater, cerebrospinal fluid leakage, sensory and dyskinesia of lower extremities, or bowel dysfunction. Regarding the time of surgery, in 1 case, the residual catheter was removed immediately after being broken, and in 6 cases, it was removed within 1 week after being broken.

The patients had no obvious low back pain after surgery, with VAS scores from 0 to 2. Stage I healing of the incision was observed, with no complications, such as infection of the incision. Before removal of the ruptured catheter, the patient had obvious anxiety. There were 5 cases of moderate anxiety and 2 case of severe anxiety. However, the postoperative patient anxiety score was significantly improved, as the patient was without anxiety. The duration of experience in anesthesiology was 21.57 ± 6.754 (12–31) months; the anesthesiologists had mild and moderate anxiety before the catheter was removed. After the catheter was removed, the patient recovered smoothly, and anxiety symptoms disappeared (Table 2).

**Table 2:** Anxiety scale ($\bar{x}\pm s$, *n* = 7**).**

	Sex (male)	Sex (female)	Preoperative SAS score	1 W Postoperative SAS score	1 M Postoperative SAS score
Anesthesiologist	5	2	59.0 ± 4.761	49.57 ± 1.512*	46.0 ± 1.155**
Patient	3	4	61.29 ± 5.345	48.86 ± 3.132*	45.86 ± 1.773**
*P*			0.4147	0.5968	0.8612

There was no significant difference in preoperative and postoperative self-rating anxiety scale between anesthesiologists and patients, *P* > 0.05.

* Compared with preoperative, *P* < 0.05.

** Compared with 1week postoperative, *P* < 0.05. SAS, Self-Rating Anxiety Scale; Y, year; M, month; W, week.

## Discussion

Epidural catheter rupture is one of the complications of epidural block in clinical anesthesia. Most patients have no obvious symptoms or signs in the early stage (1, 5, 13). But long-term retention will result in the formation of immune complex or inflammatory granuloma around the foreign material (6, 14). It is recommended to remove the residual catheter as soon as possible (7, 8). Because the surgical difficulty and risk are significantly increased while the patient shows symptoms of nerve compression, irritation, or foreign body stress response. In conventional surgery, it is necessary to remove the lamina, spinous processes, and articular processes for ensuring the position of the broken catheter, which destroys the spinal stability and requires additional internal fixation (4, 9, 15). An open approach is not only more invasive but can be very challenging if the position of the catheter cannot be adequately identified. In those circumstances only the use of Intraoperative Ultrasound (IoUS) can effectively help in reducing manipulation and traumatism of the soft tissues and neural structures (16). These factors will inevitably lead to complaints from the patients and their families, who may resort to law and initiate a medical dispute (17). Such events also obviously affect the daily work of anesthesiologists, leading to anxiety and frustration, which are not conducive to the growth and development of novice anesthesiologists.

In recent years, the level of percutaneous full-endoscopic technique (PFET) in the diagnosis and treatment of spinal diseases has improved significantly (18, 19). In this study, PFET was used to safely remove the residual epidural catheter, with less trauma. Epidural catheter rupture has a very low incidence and mostly occurs at primary hospitals. Our hospital admitted a total of 7 cases in 4 years from 7 different departments; accidents are more common among novice people who have been employed for approximately 2 years as anesthesiologists. The SAS scores of both the patient and doctor significantly decreased, and a positive and healthy outlook was obtained. In anesthesia by epidural block, a channel is created for the injection of local anesthetics (20). It can also be used for lumbar cistern drainage in neurosurgery patients (21). Epidural catheters are even used during the infusion of cryoprecipitate and platelets into the amniotic cavity to treat premature rupture of fetal membrane and oligohydramnios in pregnant women (22, 23). This approach provides a new perspective on the use of epidural catheters, prompting clinicians to understand the application and clinical value of epidural catheters and better use this clinical device (23–25). An endoscopic approach make sense because it would minimize the postoperative risk of CSF leak and intracranial hypotension than might result from incidental dural tears. This has been widely demonstrated in large series [such as those from Soma et al. which would complement well Ref. (17) from your article] where a full endoscopic technique was considered to perform discectomies and laminectomies (26).

How to minimize the risks and complications of anesthesia is an eternal topic (1, 4, 7, 8). According to the relevant literature and subjective experience, the following points merit attention: First, when it is difficult to place the epidural puncture catheter, care should be taken to remove the epidural catheter together with the puncture needle and avoid withdrawal of the tube first and then the needle. Second, it is recommended to use a reinforced epidural catheter when inserting a catheter for epidural block. Third, the catheter and the puncture needle arc should be kept close during puncture of the epidural space to minimize the possibility of the puncture cannula damaging the catheter during tube placement (27). Fourth, when removing the epidural catheter at the end of the operation, the applied force should be moderate and stable, and the catheter should not be forcibly removed (28). Fifth, when catheter removal is difficult, the patient should be assisted in returning to the puncture position for trial removal, and a deep vein puncture needle should be used to remove the epidural catheter if necessary (29). Sixth, the operator should not panic if the epidural catheter ruptures and partially remains in the body; the family of the patient should be informed in a timely manner, and the spinal surgeon or neurosurgeon specializing in spinal endoscopy should be contacted for the removal operation, or the operator should wait for the patient to stabilize and then perform endoscopic surgery to remove the broken catheter (30). Seventh, it is necessary to grasp the relevant knowledge and the process of the diagnosis and treatment of foreign bodies in the spinal canal to minimize the medical risks, iatrogenic injuries and risks for complications to ensure the safety and normal practice of clinical medicine.

The PFET removal of a residual epidural catheter is of great clinical significance. The following points should be grasped when implementing the PFET: (1) Localization of residual tube: Accurate localization is the basis of a successful operation. A detailed understanding of the previous operation and the length of the residual tube is essential for judging the location of the broken end of the catheter in the body. (2) CT and MRI examination of the lumbar spine: When using an ordinary epidural catheter, the remaining catheter is difficult to identify and can only be determined according to the specific conditions of the anesthesiologist's operation. If an enhanced epidural catheter is used, CT and MRI examinations can allow identification of the residual catheter. (3) Positioning of the surgical path based on the clear puncture gap, direction of the catheter, and length of the residual tube. Correctly determining the location of the residual broken tube in the body and applying the correct method to locate the residual tube are keys to the success of the operation. As shown in this study, PFET could successfully performed with minimal trauma, high success rate, and easy reception. It can relieve patients' anxiety, improve patients' physical and mental health, and help prevent medical disputes.

### Limitation

The shortcomings of this study are the small number of cases and the lack of cases involving the thoracic spinal canal, long-term residual catheterization and granuloma formation around residual catheters. More clinical studies are needed.

## Conclusions

PFET is a safe and effective minimally invasive technique for removing residual epidural catheters. It causes less trauma and less bleeding, allows a faster recovery. It does not affect the recovery of patients from other surgical operations and reduces both medical risks and medical costs. At the same time, it avoids or reduces the occurrence of medical disputes and eliminates the pressure on novice anesthesiologists regarding similar adverse events, and helps relieve novice anesthesiologists' professional anxiety and promotes their active and healthy work.

## Data Availability

The original contributions presented in the study are included in the article/Supplementary Material, further inquiries can be directed to the corresponding author/s.

## References

[1] Sara KT Sara A. Anesthesia management after inadvertent dural puncture during application of epidural blockage. dicle Med J. (2010) 37(4):394–6. 10.1016/j.jpainsymman.2010.06.024

[2] Cappiello E O'Rourke N Segal S Tsen LC. A randomized trial of dural puncture epidural technique compared with the standard epidural technique for labor analgesia. Anesth Analg. (2008) 107(5):1646–51. 10.1213/ane.0b013e318184ec14 18931227

[3] Koning MV Veerkamp RAM. Dual epidural catheters for acute pain management of a patient with rib and tibial plateau fractures. J Clin Anesth. (2019) 52:53–4. 10.1016/j.jclinane.2018.09.002 30196090

[4] Souza MA Silva JL Maia FN. Combined spinal-epidural block versus continuous epidural block in labor analgesia for primiparous women: newborns and women outcomes. Rev Bras Ginecol Obstet. (2009) 31(10):485–91. 10.1590/s0100-72032009001000003 19942995

[5] Anwari JS Al Wahbi Y Al Nahdi S. A broken catheter in the epidural space. Neurosciences (Riyadh). (2014) 19(2):138–41. 10.1016/j.annfar.2010.11.023 24739413

[6] Reston SC Mcdonald DA Seigmeth R Deakin AH Scott NB Kinninmonth A. Epidural catheter rupture using the caledonian technique - a cautionary note!. Bone Joint J. (2011) 93-B(SUPP I):63.

[7] Kwofie KM. Complications of regional anesthesia: principles of safe practice in local and regional anesthesia – third edition. *Can J Anesth/J Can Anesth*. (2018): s12630-018-1117-z. 10.1007/s12630-018-1117-z

[8] BenDavid B. Complications of regional anesthesia: an overview. Anesthesiol Clin North America. (2002) 20(3):665–7. 10.1016/S0889-8537(02)00003-2 12298311

[9] Mitra R Fleischmann K. Management of the sheared epidural catheter: is surgical extraction really necessary?. J Clin Anesth. (2007) 19(4):310–4. 10.1016/j.jclinane.2006.11.005 17572331

[10] Hajnour MS Khokhar RS Ejaz AA Al Zahrani T Kanchi NU. Difficulty in the removal of epidural catheter for labor analgesia. Saudi J Anesth. (2017) 11(1):117–9. 10.4103/1658-354X.197353 PMC5292835 28217071

[11] Zhou C Zhang G Panchal RR Ren X Xiang H Xue M Unique complications of percutaneous endoscopic lumbar discectomy and percutaneous endoscopic interlaminar discectomy. Pain Physician. (2018) 21(1):E105–12. 29565953

[12] Chen Z Zhang L Dong J Xie P Liu B Chen R Percutaneous transforaminal endoscopic discectomy versus microendoscopic discectomy for lumbar disc herniation: two-year results of a randomized controlled trial. Spine. (2020) 45:493–503. 10.1097/BRS.0000000000004468 31703056

[13] Blanchard N Clabeau JJ Ossart M Dekens J Legars D Tchaoussoff J. Radicular pain due to a retained fragment of epidural catheter. Anesthesiology. (1997) 87:1567–9. 10.1097/00000542-199712000-00036 9416742

[14] Pierre HL Block BM Wu CL. Difficult removal of a wire- reinforced epidural catheter. J Clin Anesth. (2003) 15(2):140–1. 10.1016/S0952-8180(02)00516-0 12719055

[15] Tarukado K Oda T Tono O Suetsugu H Doi T. A retained epidural catheter fragment treated by surgery. Asian Spine J. (2015) 9(3):461–4. 10.4184/asj.2015.9.3.461 26097665 PMC4472598

[16] Ganau M Syrmos N Martin AR Jiang F Fehlings MG. Intraoperative ultrasound in spine surgery: history, current applications, future developments. Quant Imaging Med Surg. (2018) 8(3):261–7. 10.21037/qims.2018.04.02 29774179 PMC5941206

[17] Carneiro S Pinto C. Sheared epidural catheter: what now?. Eur J Anaesthesiol. (2013) 30:125–125. 10.1097/00003643-201306001-00389

[18] Lee CH Choi M Ryu DS Choi I Kim CH Kim HS Efficacy and safety of full-endoscopic decompression via interlaminar approach for central or lateral recess spinal stenosis of the lumbar spine. Spine. (2018) 1:1756–64. 10.1097/BRS.0000000000002708 29794584

[19] Sebastian R Hahn P Oezdemir S Baraliakos X Godolias G Komp M. Decompression of the anterior thoracic spinal canal using a novel full-endoscopic uniportal transthoracic retropleural technique-an anatomical feasibility study in human cadavers. Clin Anat. (2018) 31(5):716–23. 10.1002/ca.23075 29577428

[20] Förster JG Niemi TT Salmenperä MT Ikonen S Rosenberg PH. An evaluation of the epidural catheter position by epidural nerve stimulation in conjunction with continuous epidural analgesia in adult surgical patients. Anesth Analg. (2009) 108(1):351–8. 10.1213/ane.0b013e31818d0392 19095872

[21] Cesur M Alici HA Erdem AF Silbir F Yuksek MS. Administration of local anesthetic through the epidural needle before catheter insertion improves the quality of anesthesia and reduces catheter-related complications. Anesth Analg. (2005) 101(5):1501–5. 10.1213/01.ANE.0000181005.50958.1E 16244020

[22] Manchikanti L Soin A Mann DP Bakshi S Pampati V Hirsch JA. Comparative analysis of utilization of epidural procedures in managing chronic pain in the medicare population. Spine. (2019) 44(3):220–32. 10.1097/BRS.0000000000002785 30005043

[23] Lange EMS Wong CA Fitzgerald PC Davila WF Rao S McCarthy RJ Effect of epidural infusion bolus delivery rate on the duration of labor analgesia. Anesthesiology. (2018) 1:745–53. 10.1097/ALN.0000000000002089 29351097

[24] Hu A Gu X Guan X Fan G He S. Epidural versus intravenous steroids application following percutaneous endoscopic lumbar discectomy. Medicine (Baltimore). (2018) 97(18):e0654. 10.1097/MD.0000000000010654 29718884 PMC6392748

[25] Turgut A Katar S Sak ME Turgut FG Sahin A Başaranoğlu S Continuous amnioinfusion via an epidural catheter following spontaneous membrane rupture: a case report. J Turk Ger Gynecol Assoc. (2013) 14(4):238–41. 10.5152/jtgga.2013.53367 24592114 PMC3935536

[26] Soma K Kato S Oka H Matsudaira K Fukushima M Oshina M Influence of incidental dural tears and their primary microendoscopic repairs on surgical outcomes in patients undergoing microendoscopic lumbar surgery. Spine J. (2019) 19(9):1559–65. 10.1016/j.spinee.2019.04.015 31009767

[27] Gravenstein N Blackshear RH Wissler RN. An approach to spinal or epidural catheters that are difficult to remove. Anesthesiology. (1991) 75(3):544. 10.1097/00000542-199109000-00030 1741861

[28] Ugboma S Au Truong X Kranzler LI Rifai SH Joseph NJ Salem MR. The breaking of an intrathecally-placed epidural catheter during extraction. Anesth Analg. (2002) 95(4):1087–9. 10.1213/00000539-200210000-00055 12351300

[29] Morris GN Warren BB Hanson EW Mazzeo FJ DiBenedetto DJ. Influence of patient position on withdrawal forces during removal of lumbar extradural catheters. Br J Anaesth. (1996) 77(3):419–20. 10.1093/bja/77.3.419 8949823

[30] Noblett K McKinney A Kim R. Sheared epidural catheter during an elective procedure. Obstet Gynecol. (2007) 109(2 Pt2):566–8. 10.1097/01.AOG.0000253246.56575.84 17267897

